# Trajectory of low-density lipoprotein cholesterol in patients with chronic kidney disease and its association with cardiovascular disease

**DOI:** 10.3389/fcvm.2022.887915

**Published:** 2022-07-26

**Authors:** Shih-Wei Wang, Lung-Chih Li, Chung-Ming Fu, Yueh-Ting Lee, Hsiao-Ching Kuo, Chien-Ning Hsu

**Affiliations:** ^1^Department of Pharmacy, Kaohsiung Chang Gung Memorial Hospital, Kaohsiung, Taiwan; ^2^School of Pharmacy, Kaohsiung Medical University, Kaohsiung, Taiwan; ^3^Division of Nephrology, Department of Internal Medicine, Kaohsiung Chang Gung Memorial Hospital and Chang Gung University College of Medicine, Kaohsiung, Taiwan; ^4^Institute for Translational Research in Biomedicine, Kaohsiung Chang Gung Memorial Hospital and Chang Gung University College of Medicine, Kaohsiung, Taiwan

**Keywords:** chronic kidney disease, low-density lipoprotein cholesterol (LDL-C), cardiovascular disease, trajectory, diabetes

## Abstract

**Background:**

The role of longitudinal temporal trends in LDL-C in cardiovascular disease (CVD) in patients with chronic kidney disease (CKD) and diabetes is unclear. This study categorized the long-term LDL-C trajectory and determined its association with the incidence of atherosclerotic CVD in patients with CKD according to diabetes status and estimated glomerular filtration rate (eGFR).

**Methods:**

The risk of atherosclerotic CVD was estimated in 137,127 Taiwanese patients with CKD using six LDL-C trajectory classes determined by the latent class mixed model as optimal, near optimal, above optimal, borderline, sustained high, and declined high over 5 years.

**Results:**

The risk of CVD was higher in the sustained high LDL-C [>160 mg/dL over time; adjusted hazard ratio (aHR) = 1.68, 95% CI = 1.45–1.94], declined high LDL-C (>160 to <100 mg/dL; aHR = 1.23, 95% CI = 1.11–1.38), and borderline LDL-C (approximately 140 mg/dL over time; aHR = 1.16, 95% CI = 1.07–1.26) groups than in the optimal LDL-C group (<100 mg/dL over time). There was no such association in patients with an eGFR <15 mL/min/1.73 m^2^. Persistent diabetes was associated with a 1.15–2.47-fold increase in CVD in patients with high LDL-C (>120 mg/dL).

**Conclusion:**

The LDL-C trajectory pattern was associated with the phenotype of CVD risk. The degree of risk varied according to eGFR and diabetes status. A stable low LDL-C over time was potentially beneficial for prevention of CVD. Intensive lipid management and periodic assessment of LDL-C is essential to reduce the risk of CVD in patients with CKD and diabetes.

## Introduction

Cardiovascular (CV) morbidity and mortality is high in patients with chronic kidney disease (CKD) (1), and atherosclerosis-associated CV events are more likely to be fatal in these patients (2). However, lipid abnormalities are not consistent between the various etiological categories of CKD (e.g., with or without proteinuria) or glomerular filtration rate (3). Therefore, the age-related changes in LDL-C concentration associated with an increased risk of cardiovascular disease (CVD) in the general population (4) may not be generalizable to patients with CKD.

The 2013 Kidney Disease: Improving Global Outcomes (KDIGO) Clinical Practice Guideline recommends prescribing a statin or statin/ezetimibe in adults aged ≥50 years with an estimated glomerular filtration rate (eGFR) <60 mL/min/1.73 m^2^ but does not mention specific LDL-C targets to initiate treatment with a statin or increase the statin dose nor repeated measurement of LDL-C (5). Despite the trial-setting evidence of the novel lipid-lowering agents cited in the KDIGO guideline being warranted for appraisal in the real-world population (6–8), the lipophilic or hydrophilic nature of statins may affect their ability to prevent CV events in patients with dialysis-dependent CKD (9). Furthermore, the current evidence is based on trials that focused on the baseline LDL-C level and does not take dynamic changes in the LDL-C level into consideration. Changes in the LDL-C concentration can result from lifestyle modification and adherence with lipid-lowering therapies over time (10) with variable results in terms of the risk of atherosclerotic CVD (11, 12).

Several studies have found that the longitudinal trajectories of lipids are heterogeneous in the general population (4, 13, 14); however, there is limited relevant information in the CKD population, resulting in a knowledge gap concerning risk management in this group. In this study, we aimed to identify the association of the LDL-C trajectory with incident CVD and to determine the clinical factors related to variations in changes in LDL-C concentration and their association with development of atherosclerotic CVD according to diabetes status.

## Materials and methods

### Data sources

The CKD cohort used in this study was identified using the Chang Gung Research Database (CGRD), which is an electronic health record derived from the Chang Gung Memorial Hospital network in Taiwan. This network, with a total of 9,584 beds, delivered approximately 11% of health care services reimbursed by Taiwan’s national health insurance program in 2018, including over 9.1 million emergency and outpatient department visits and 300,000 hospital admissions (15). The CGRD contains detailed information on diagnoses, prescriptions, examinations, and laboratory test results in both inpatient and outpatient settings. The study was approved by the Institutional Review Board of the Chang Gung Medical Foundation in Taipei, Taiwan (permit number 201900900B0C501).

### Study cohort

The study cohort consisted of patients with a new diagnosis of CKD between January 1, 2004 and December 31, 2018. CKD was confirmed using the International Classification of Diseases, Ninth/Tenth Revision, Clinical Modification (ICD-9/10-CM) for CKD with ≥1 ICD-9/10 code for inpatients or at least two ICD-9/10 codes for CKD in 1 year and ≥3 months apart for outpatients (16). The mean eGFR was calculated every 3 months to avoid misclassification of CKD, and patients who started with a mean eGFR <60 mL/min/1.73 m^2^ were identified as having incident CKD. The date of diagnosis of CKD was assigned as the date with ICD-9/10 codes for CKD or the date with a mean eGFR <60 mL/min/1.73 m^2^, whichever came first.

To assess the longitudinal trajectory of LDL-C in the CKD population, patients were excluded if they had (1) fewer than three measurements of LDL-C during the study follow-up period and (2) a time interval between the first and last LDL-C measurement of less than 1 year. To avoid missing baseline serum creatinine values, patients were excluded if there was no any medical encounters before the index date. For estimation of the incident risk of CVD, patients who had been diagnosed with myocardial infarction (MI), unstable angina, or ischemic stroke and those who underwent coronary artery bypass grafting or percutaneous coronary intervention were also excluded.

### Outcomes

The primary outcome was the composite atherosclerotic CV endpoint of MI, ischemic stroke, and unstable angina. Secondary outcomes included MI, ischemic stroke, and unstable angina. The onset of outcome events during follow-up was identified using the ICD-9/10 code at hospital discharge or outpatient diagnosis. The index date was defined as the date of the first LDL measurement. All patients were followed up from the index date until the outcome event of interest, death, loss to follow-up, or the latest date in the dataset (December 31, 2018), whichever came first.

### Analysis of LDL-C trajectory

LDL-C levels were assessed within a 1-year window over the study period. The annual time-averaged LDL-C level was employed to some patients who had more than one LDL-C measurement in 1-year window (17, 18). Patients with repeated LDL-C levels over the study period were included in LDL-C trajectory analysis, which will be described in the section of “statistical” analysis. We named the six trajectory categories according to LDL-C levels using the National Cholesterol Education Program Adult Treatment Panel III guidelines as follows: optimal, <100 mg/dL; near/above optimal, 100–129 mg/dL; borderline high, 130–159 mg/dL; high, 160–189 mg/dL, and very high, ≥190 mg/dL (19). Given that there is no gold standard LDL-C target in the CKD population, the LDL-C cut-off level was data-driven based on the performance of the latent class model, details of which are described in the statistical analysis.

### Covariates

Information was collected on baseline characteristics, including age, sex, eGFR, CKD stage, baseline LDL-C, comorbidities (Charlson Comorbidity Index (20), hypertension, and dyslipidemia), and medications (lipid-lowering agents, antidiabetic agents, antihypertensive agents, antiplatelets, anticoagulants) within 365 days before the index date. Use of concomitant medication during follow-up (before the event of interest or end-of-follow-up date) was identified in the outpatient setting. The medication usage was defined as the medication category has been prescribed more than 28 cumulative defined daily dose (cDDD) (21) within 365 days before the index date and during follow-up period.

### Statistical analysis

#### Categorization of LDL-C trajectories

A latent class growth mixed model (LCGMM) was used to characterize the trajectories of LDL-C levels during the study period of 5 years using the SAS function “Proc traj” (22). SAS Proc traj censored normal (CNORM) model without covariates was applied to identify LDL-C trajectories (Supplementary Table 1). To identify the “best-fit” model, several models with ranging from 3 to 7 groups with polynomials of varying degrees in each group were assessed. The number of trajectory groups was determined by balancing clinical knowledge against the criteria as follows: (1) lowest Bayesian information criterion value (22); (2) average posterior probability highest and above 0.7 for all latent classes (j = 1,…*J*, where *J* is the number of trajectory groups) (23); and (3) no less than 1.5% of participants in any single trajectory group (4). The odds of correct classification (OCC) >5 for all LDL-C trajectory groups were also applied to examine the mode fit (23). A linear mixed model was used to identify annual changes in LDL-C concentration over time in each trajectory group and adjusted for baseline patient characteristics.

#### Trajectories of LDL-C and incident cardiovascular disease

The crude cumulative incidence rate of CVD was presented with percentage and 1,000 person-year by the groups of LDL-C trajectory. The Chi-square test was used to examine the distribution of CVD event rate among groups. Associations between the lipid trajectory and CV outcomes were estimated using a Cox proportional hazard regression model with adjustment for baseline characteristics and uses of concomitant medications (as a time-varying confounding factor). Factors associated with the longitudinal variability of LDL-C during follow-up, such as diabetes mellitus and baseline eGFR, were examined in the stratified analyses to evaluate the heterogeneity of the association between LDL-C trajectory and CVD risk in different subpopulations. In addition, weighted Cox proportional hazard model was generated based on each person’s posterior probability in the specific trajectory group and a robust sandwich estimator was used in the model to account for intraperson correlation (14). Same covariates were adjusted in all Cox regression models so that results across were more comparable. All analyses were performed using SAS software (version 9.4; SAS Institute Inc., Cary, NC, United States). A two-sided *P*-value < 0.05 was considered statistically significant.

## Results

### Patient characteristics

The study cohort included 137,127 patients with newly diagnosed CKD and at least three LDL-C measurements who met the inclusion and exclusion criteria (Supplementary Figure 1). Baseline characteristics according to six distinct LDL-C trajectory groups based on the National Cholesterol Education Program Adult Treatment Panel III guideline are shown in Table 1. The mean age was 61.73 ± 13.26 years, and 52.43% of the study cohort were male. The mean baseline LDL-C concentration was 112.87 ± 36.23 mg/dL. Among 62,064 patients had less optimal LDL trajectory patterns (above optimal, borderline, sustained high, and declined high), 16% (9,949) were treated with lipid-lowering agent at baseline.

**TABLE 1:** Baseline characteristics of the chronic kidney disease cohort by LDL-C trajectory class and baseline risk category.

Variables	Overall (*n* = 137,127)	Optimal	Near optimal	Above optimal	Borderline	Sustained high	Declined high
							
		(LDL-C < 100) (*n* = 21,120)	(LDL-C near 100) (*n* = 53,943)	(LDL-C near 120) (*n* = 42,059)	(LDL-C near 140) (*n* = 14,014)	(LDL-C > 160) (*n* = 2,113)	(LDL-C > 160 to near 100) (*n* = 3,878)
**Sex, *n* (%)**							
Male	71,890	11,843 (56.07)	28,357 (52.57)	21,731 (51.67)	7,104 (50.69)	1,022 (48.37)	1,833 (47.27)
Female	65,237	9,277 (43.93)	25,586 (47.43)	20,328 (48.33)	6,910 (49.31)	1,091 (51.63)	2,045 (52.73)
**Baseline LDL-C, mg/dL, n (%)**							
<100	52,979	17,486 (82.79)	27,225 (50.47)	7,454 (17.72)	723 (5.16)	42 (1.99)	49 (1.26)
100–130	45,571	2,512 (11.89)	18,786 (34.83)	20,704 (49.23)	3,166 (22.59)	121 (5.73)	282 (7.27)
>130	38,577	1,122 (5.31)	7,932 (14.70)	13,901 (33.05)	10,125 (72.25)	1,950 (92.29)	3,547 (91.46)
**Baseline eGFR, mL/min/1.73 m^2^, *n* (%)**							
≧90 (1)	26,284	4,254 (20.14)	10,895 (20.20)	7,767 (18.47)	2,591 (18.49)	325 (15.38)	452 (11.66)
60–89.9 (2)	51,596	8,144 (38.56)	20,660 (38.30)	15,774 (37.50)	5,105 (36.43)	705 (33.36)	1,208 (31.15)
45–59.9 (3a)	35,276	4,628 (21.91)	13,010 (24.12)	11,627 (27.64)	4,138 (29.53)	669 (31.66)	1,204 (31.05)
30–44.9 (3b)	12,573	2,011 (9.52)	4,956 (9.19)	3,781 (8.99)	1,115 (7.96)	197 (9.32)	513 (13.23)
15–29.9 (4)	6,011	1,061 (5.02)	2,405 (4.46)	1,633 (3.88)	497 (3.55)	111 (5.25)	304 (7.84)
<15 (5)	5,387	1,022 (4.84)	2,017 (3.74)	1,477 (3.51)	568 (4.05)	106 (5.02)	197 (5.08)
**CCI, *n* (%)**							
Congestive heart failure	4,656	877 (4.15)	1,969 (3.65)	1,279 (3.04)	358 (2.55)	45 (2.13)	128 (3.30)
Peripheral vascular diseases	1,770	349 (1.65)	755 (1.40)	479 (1.14)	122 (0.87)	20 (0.95)	45 (1.16)
Cerebral vascular accident	1,879	363 (1.72)	773 (1.43)	530 (1.26)	131 (0.93)	17 (0.80)	65 (1.68)
Dementia	1,556	324 (1.53)	667 (1.24)	398 (0.95)	116 (0.83)	12 (0.57)	39 (1.01)
Pulmonary disease	9,093	1,569 (7.43)	3,661 (6.79)	2,661 (6.33)	821 (5.86)	111 (5.25)	270 (6.96)
Connective tissue disorder	1,399	176 (0.83)	495 (0.92)	439 (1.04)	191 (1.36)	35 (1.66)	63 (1.62)
Peptic ulcer	14,750	2,578 (12.21)	5,927 (10.99)	4,336 (10.31)	1,317 (9.40)	198 (9.37)	394 (10.16)
Liver diseases	18,457	3,424 (16.21)	7,488 (13.88)	5,290 (12.58)	1,632 (11.65)	239 (11.31)	384 (9.90)
Diabetes	49,391	10,859 (51.42)	22,674 (42.03)	11,816 (28.09)	2,708 (19.32)	358 (16.94)	976 (25.17)
Diabetes complications	12,555	2,917 (13.81)	5,703 (10.57)	2,759 (6.56)	698 (4.98)	120 (5.68)	358 (9.23)
Paraplegia	331	68 (0.32)	121 (0.22)	103 (0.24)	24 (0.17)	3 (0.14)	12 (0.31)
Renal disease	14,093	2,009 (9.51)	4,901 (9.09)	4,604 (10.95)	1,736 (12.39)	299 (14.15)	544 (14.03)
Cancer	8,967	1,619 (7.67)	3,560 (6.60)	2,544 (6.05)	861 (6.14)	159 (7.52)	224 (5.78)
Severe liver diseases	467	161 (0.76)	186 (0.34)	79 (0.19)	19 (0.14)	5 (0.24)	17 (0.44)
Metastatic cancer	736	163 (0.77)	268 (0.50)	207 (0.49)	72 (0.51)	11 (0.52)	15 (0.39)
Hypertension	64,798	11,510 (54.50)	27,281 (50.57)	18,632 (44.30)	5,080 (36.25)	610 (28.87)	1,685 (43.45)
Dyslipidemia	48,173	8,959 (42.42)	20,619 (38.22)	13,030 (30.98)	3,822 (27.27)	551 (26.08)	1,192 (30.74)
**Baseline medication, *n* (%)**							
**Lipid-lowering agents**	28,782	5,912 (27.99)	12,921 (23.95)	7,071 (16.81)	1,922 (13.71)	322 (15.24)	634 (16.35)
Statins	22,194	4,704 (22.27)	9,966 (18.48)	5,261 (12.51)	1,509 (10.77)	278 (13.16)	476 (12.27)
Fibrates	7,655	1,422 (6.73)	3,493 (6.48)	2,076 (4.94)	436 (3.11)	48 (2.27)	180 (4.64)
Others	4,405	1,107 (5.24)	1,864 (3.46)	956 (2.27)	333 (2.38)	69 (3.27)	76 (1.96)
**Antidiabetic agents**	51,282	11,614 (54.99)	23,667 (43.87)	11,984 (28.49)	2,662 (19.00)	352 (16.66)	1,003 (25.86)
Insulin	7,688	1,758 (8.32)	3,391 (6.29)	1,772 (4.21)	479 (3.42)	81 (3.83)	207 (5.34)
Metformin	42,228	9,781 (46.31)	19,793 (36.69)	9,659 (22.97)	2,010 (14.34)	257 (12.16)	728 (18.77)
SGLT2 inhibitors	308	94 (0.45)	144 (0.27)	48 (0.11)	13 (0.09)	7 (0.33)	2 (0.05)
DPP4 inhibitors	11,098	2,867 (13.57)	5,185 (9.61)	2,352 (5.59)	493 (3.52)	56 (2.65)	145 (3.74)
Sulfonylureas	20,986	4,358 (20.63)	9,470 (17.56)	5,291 (12.58)	1,182 (8.43)	159 (7.52)	526 (13.56)
Acarbose	7,706	1,764 (8.35)	3,453 (6.40)	1,867 (4.44)	431 (3.08)	55 (2.60)	136 (3.51)
Thiazolidinediones	5,993	1,456 (6.89)	2,853 (5.29)	1,240 (2.95)	260 (1.86)	41 (1.94)	143 (3.69)
		(LDL-C < 100) (*n* = 21,120)	(LDL-C near 100) (*n* = 53,943)	(LDL-C near 120) (*n* = 42,059)	(LDL-C near 140) (*n* = 14,014)	(LDL-C > 160) (*n* = 2,113)	(LDL-C > 160 to near 100) (*n* = 3,878)
GLP1 receptor agonist	209	65 (0.31)	81 (0.15)	44 (0.10)	14 (0.10)	4 (0.19)	1 (0.03)
Meglitinides	3,290	692 (3.28)	1,416 (2.62)	835 (1.99)	205 (1.46)	29 (1.37)	113 (2.91)
**Antihypertensive agents**	72,039	12,769 (60.46)	29,793 (55.23)	20,983 (49.89)	5,751 (41.04)	753 (35.64)	1,990 (51.32)
ACEI	9,123	1,606 (7.60)	3,759 (6.97)	2,558 (6.08)	762 (5.44)	99 (4.69)	339 (8.74)
ARB	47,057	8,877 (42.03)	20,009 (37.09)	13,222 (31.44)	3,361 (23.98)	435 (20.59)	1,153 (29.73)
Direct renin inhibitor	818	182 (0.86)	325 (0.60)	230 (0.55)	58 (0.41)	6 (0.28)	17 (0.44)
Diuretics	20,132	3,531 (16.72)	8,028 (14.88)	5,798 (13.79)	1,684 (12.02)	313 (14.81)	778 (20.06)
Beta-blockers	27,890	4,888 (23.14)	11,495 (21.31)	8,219 (19.54)	2,214 (15.80)	272 (12.87)	802 (20.68)
Calcium channel blockers	34,886	6,033 (28.57)	14,241 (26.40)	10,324 (24.55)	2,885 (20.59)	353 (16.71)	1,050 (27.08)
**Antiplatelets**	20,878	4,519 (21.40)	9,079 (16.83)	5,262 (12.51)	1,308 (9.33)	178 (8.42)	532 (13.72)
Aspirin	17,912	3,951 (18.71)	7,878 (14.60)	4,442 (10.56)	1,078 (7.69)	129 (6.11)	434 (11.19)
Clopidogrel	1,584	372 (1.76)	696 (1.29)	379 (0.90)	91 (0.65)	12 (0.57)	34 (0.88)
Ticagrelor	7	3 (0.01)	3 (0.01)	1 (0.00)	0 (0.00)	0 (0.00)	0 (0.00)
Other Anti-platelet	2,696	483 (2.29)	1,051 (1.95)	790 (1.88)	223 (1.59)	48 (2.27)	101 (2.60)
**Anticoagulants**	1,919	443 (2.10)	823 (1.53)	497 (1.18)	113 (0.81)	14 (0.66)	29 (0.75)
Warfarin	1,548	335 (1.59)	668 (1.24)	410 (0.97)	100 (0.71)	13 (0.62)	22 (0.57)
Dabigatran	199	69 (0.33)	83 (0.15)	38 (0.09)	5 (0.04)	1 (0.05)	3 (0.08)
Rivaroxaban	229	60 (0.28)	92 (0.17)	64 (0.15)	8 (0.06)	1 (0.05)	4 (0.10)
Apixaban	9	2 (0.01)	6 (0.01)	1 (0.00)	0 (0.00)	0 (0.00)	0 (0.00)
Edoxaban	4	1 (0.00)	3 (0.01)	0 (0.00)	0 (0.00)	0 (0.00)	0 (0.00)

**Mean (*SD*)**							

Age at the index date, year	61.73 (13.26)	64.04 (13.13)	62.78 (12.97)	60.87 (13.24)	58.17 (13.27)	55.31 (13.76)	60.20 (13.38)
Baseline eGFR, ml/min/1.73 m^2^	67.58 (29.46)	67.50 (30.41)	68.30 (29.67)	67.62 (28.86)	67.54 (28.93)	63.98 (29.65)	59.56 (28.01)
**Medication use during follow-up, *n* (%)**							
**Lipid-lowering agents**	69,520	10,472 (49.58)	27,762 (51.47)	20,144 (47.89)	7,166 (51.13)	1,256 (59.44)	2,720 (70.14)
Statins	60,636	8,808 (41.70)	23,913 (44.33)	17,584 (41.81)	6,583 (46.97)	1,183 (55.99)	2,565 (66.14)
Fibrates	17,115	2,616 (12.39)	7,456 (13.82)	5,165 (12.28)	1,240 (8.85)	176 (8.33)	462 (11.91)
Others	16,296	2,134 (10.10)	5,567 (10.32)	4,917 (11.69)	2,250 (16.06)	531 (25.13)	897 (23.13)
**Anti-diabetic agents**	72,613	14,845 (70.29)	32,586 (60.41)	18,331 (43.58)	4,430 (31.61)	652 (30.86)	1,769 (45.62)
Insulin	16,833	3,496 (16.55)	7,363 (13.65)	4,160 (9.89)	1,063 (7.59)	204 (9.65)	547 (14.11)
Metformin	60,123	12,464 (59.02)	27,400 (50.79)	14,978 (35.61)	3,525 (25.15)	465 (22.01)	1,291 (33.29)
SGLT2 inhibitors	8,613	1,932 (9.15)	4,023 (7.46)	1,962 (4.66)	500 (3.57)	58 (2.74)	138 (3.56)
DPP4 inhibitors	29,472	6,299 (29.82)	13,335 (24.72)	7,241 (17.22)	1,620 (11.56)	246 (11.64)	731 (18.85)
Sulfonylureas	33,301	6,532 (30.93)	14,689 (27.23)	8,644 (20.55)	2,144 (15.30)	333 (15.76)	959 (24.73)
Acarbose	18,170	3,709 (17.56)	8,153 (15.11)	4,578 (10.88)	1,101 (7.86)	154 (7.29)	475 (12.25)
Thiazolidinediones	12,948	2,692 (12.75)	5,908 (10.95)	3,146 (7.48)	739 (5.27)	113 (5.35)	350 (9.03)
GLP1 receptor agonist	2,192	479 (2.27)	1,002 (1.86)	510 (1.21)	145 (1.03)	19 (0.90)	37 (0.95)
Meglitinides	8,604	1,608 (7.61)	3,664 (6.79)	2,324 (5.53)	560 (4.00)	107 (5.06)	341 (8.79)
**Anti-hypertensive agents**	103,922	16,992 (80.45)	41,936 (77.74)	31,132 (74.02)	9,357 (66.77)	1,315 (62.23)	3,190 (82.26)
ACEI	17,150	2,834 (13.42)	6,890 (12.77)	5,000 (11.89)	1,464 (10.45)	233 (11.03)	729 (18.80)
ARB	80,445	13,328 (63.11)	32,828 (60.86)	23,974 (57.00)	6,847 (48.86)	986 (46.66)	2,482 (64.00)
Direct renin inhibitor	2,977	460 (2.18)	1,145 (2.12)	881 (2.09)	283 (2.02)	65 (3.08)	143 (3.69)
Diuretics	38,559	6,330 (29.97)	15,127 (28.04)	11,272 (26.80)	3,553 (25.35)	668 (31.61)	1,609 (41.49)
Beta-blockers	50,316	8,126 (38.48)	19,974 (37.03)	15,227 (36.20)	4,551 (32.47)	640 (30.29)	1,798 (46.36)
		(LDL-C < 100) (*n* = 21,120)	(LDL-C near 100) (*n* = 53,943)	(LDL-C near 120) (*n* = 42,059)	(LDL-C near 140) (*n* = 14,014)	(LDL-C > 160) (*n* = 2,113)	(LDL-C > 160 to near 100) (*n* = 3,878)
Calcium channel blockers	60,407	9,497 (44.97)	23,916 (44.34)	18,385 (43.71)	5710 (40.74)	782 (37.01)	2117 (54.59)
**Antiplatelets**	41,191	7,418 (35.12)	17,128 (31.75)	11,500 (27.34)	3,239 (23.11)	496 (23.47)	1,410 (36.36)
Aspirin	34,602	6,254 (29.61)	14,512 (26.90)	9,638 (22.92)	2,647 (18.89)	389 (18.41)	1,162 (29.96)
Clopidogrel	6,443	1,279 (6.06)	2,707 (5.02)	1,693 (4.03)	455 (3.25)	77 (3.64)	232 (5.98)
Ticagrelor	50	10 (0.05)	24 (0.04)	13 (0.03)	2 (0.01)	0 (0.00)	1 (0.03)
Other antiplatelet	8,215	1,307 (6.19)	3,154 (5.85)	2,451 (5.83)	772 (5.51)	144 (6.81)	387 (9.98)
**Anticoagulants**	5,801	1,159 (5.49)	2,476 (4.59)	1,597 (3.80)	385 (2.75)	40 (1.89)	144 (3.71)
Warfarin	3,250	630 (2.98)	1,392 (2.58)	868 (2.06)	249 (1.78)	30 (1.42)	81 (2.09)
Dabigatran	923	212 (1.00)	409 (0.76)	238 (0.57)	44 (0.31)	3 (0.14)	17 (0.44)
Rivaroxaban	1,757	357 (1.69)	751 (1.39)	490 (1.17)	110 (0.78)	9 (0.43)	40 (1.03)
Apixaban	789	171 (0.81)	341 (0.63)	210 (0.50)	39 (0.28)	5 (0.24)	23 (0.59)
Edoxaban	595	111 (0.53)	257 (0.48)	183 (0.44)	32 (0.23)	1 (0.05)	11 (0.28)

LDL-C trajectories were categorized according to the National Cholesterol Education Program Adult Treatment Panel III levels into five levels (optimal <100, near optimal/above optimal 100–129, borderline high 130–159, high 160–189, and very high ≥190 mg/dL) (19). ACEI, angiotensin-converting enzyme inhibitors; ARB, angiotensin receptor blockers; DPP4, dipeptidyl peptidase 4; eGFR, estimated glomerular filtration rate; GLP1, glucagon-like peptide-1; SGLT2, sodium-glucose cotransporter 2.

In the groups with optimal and near-optimal LDL-C trajectories (baseline LDL-C <100 mg/dL), 25.1% of patients were treated at baseline with lipid-lowering agents, 47.0% with antidiabetic agents, and 56.7% with antihypertensive agents; these rates were higher than in the groups with a high LDL-C trajectory. Use of medications increased during the follow-up period in both the sustained high and declined high groups (baseline LDL >160 mg/dL); in particular, use of lipid-lowering agents was high in the sustained high (59.44%) and declined high (70.14%) groups, and antihypertensive agents were used in 62.23 and 82.26%, respectively (Table 1). A U-shaped distribution, that is, a relatively higher proportion of patients with advanced CKD (eGFR <30 mL/min/1.73 m^2^), was observed in patients with declined high (12.92%), sustained high (10.27%), and optimal (9.86%) LDL-C trajectories than in patients with near optimal (8.20%), above optimal (7.39%), or borderline (7.60%) trajectories (Table 1).

### Predicted LDL-C trajectories

**FIGURE 1:**
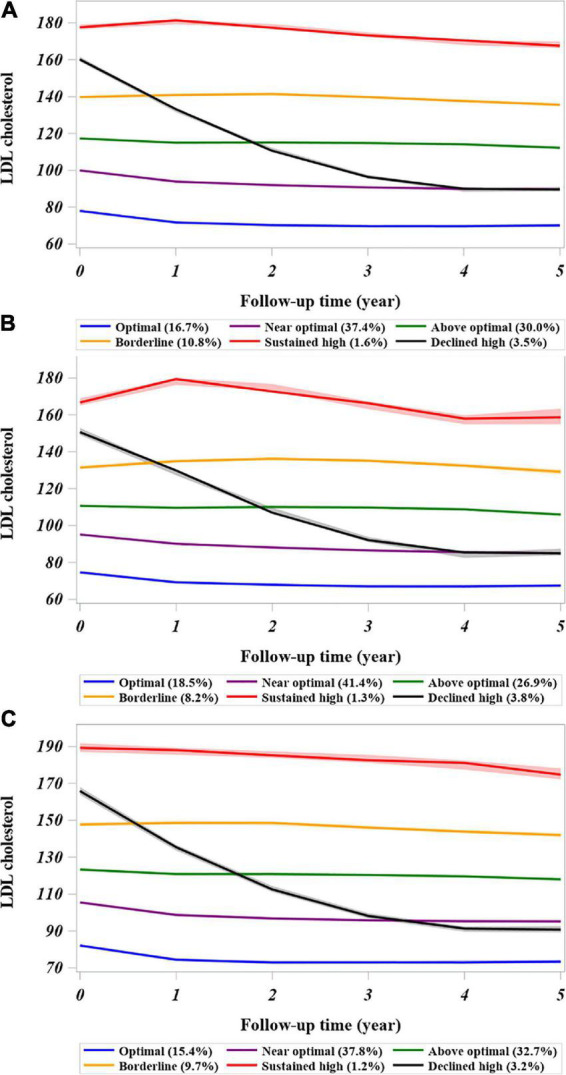
Trajectories of low-density lipoprotein-cholesterol derived from the latent class growth mixed model in the cohort of patients with CKD. **(A)** Entire chronic kidney disease cohort. **(B)** Patients with CKD and diabetes at baseline. **(C)** Patients with CKD without diabetes at baseline. The LDL-C trajectory classes were defined as follows: 1, sustained high; (baseline LDL-C >160 mg/dL that continued over time); 2, declined high (baseline LDL-C ≥160 mg/dL but declined to <100 mg/dL in the follow-up period); 3, borderline high baseline LDL-C remained in range of 130–159 mg/dL over time; 4, above optimal (baseline LDL-C around 120 mg/dL); 5, near-optimal (baseline LDL-C around 100 mg/dL); and 6, optimal (baseline LDL-C level <100 mg/dL). The LDL-C values represent the group mean with the confidence interval (shadowed). CKD, chronic kidney disease.

Based on the model selection criteria, a model of cubic parameters with 6 classes was selected from 3 to 7 classes of models investigated (Supplementary Table 1). Given that LDL-C measurements were retrieved for individual patients who had an event of interest or reached the end of follow-up, 98% of patients had at least two measurements in 5 years that could be modeled in a specific LDL-C trajectory. The mean posterior probability for individuals ranged from 0.75 to 0.88, suggesting overall good discrimination ability over 5 years of follow-up. The OCC >5 for all trajectory groups satisfied the optimal model fit criteria (Supplementary Table 2). Figure 1A depicts the LDL-C trajectories with 6 classes during follow-up in the CKD cohort. The majority of patients had a stable LDL-C trajectory over time, except for patients in the declined high group. Compared with the optimal LDL-C trajectory, the adjusted mean annual change in the LDL-C concentration was within ± 1 mg/dL in most trajectory groups but was −15.34 mg/dL (−15.50, −15.18) in the declined high group (Supplementary Table 3).

The visualized distributions of predicted mean annual change in LDL-C showed substantial overlap across most trajectory groups, but wide variations were observed in the sustained (1.53 ± 26.25 mg/dL) and declined high (−25.37 ± 19.18 mg/dL) groups (Supplementary Figure 2A). The changes in predicted mean annual LDL-C in these patients with CKD varied according to their diabetes status (Supplementary Figures 2B,C). In patients with CKD and diabetes, the annual decline in LDL-C in the above optimal group was less than that in those without diabetes (−0.68 ± 14 mg/dL vs. −1.49 ± 12.29 mg/dL). However, there was an annual increase or worsening of LDL-C in the borderline group and sustained high group (3.30 ± 16.69 mg/dL vs. 5.21 ± 33.4 mg/dL) in comparison with the patients with CKD without diabetes (0.7 ± 11.91 mg/dL vs. −0.26 ± 21.74 mg/dL).

### Association between LDL-C trajectories and cardiovascular disease outcomes

Table 2 shows the incidence of CV events in the six LDL-C trajectory groups. Overall, the composite CV event rate was 8.78% (*n* = 12,039) during the follow-up period (15.67 per 1,000 person-years). The incidence of composite CVD was significantly higher in the declined high LDL-C trajectory group (*n* = 498, 12.84%, 19.19 per 1,000 person-years), followed by the sustained high LDL-C trajectory group (*n* = 224, 10.60%, 16.96 per 1,000 person-years), in comparison with the other LDL-C trajectory groups (*p* < 0.0001).

**TABLE 2:** Incidence of cardiovascular events in the six LDL-C trajectory groups.

	Overall (*n* = 137,127)	per 1,000 PY	Optimal LDL-C < 100 (*n* = 21,120)	per 1,000 PY	Near optimal LDL-C near 100 (*n* = 53,943)	per 1,000 PY	Above optimal LDL-C near 120 (*n* = 42,059)	Per 1,000 PY	Borderline LDL-C near 140 (*n* = 14,014)	Per 1,000 PY	Sustained high LDL-C > 160 (*n* = 2,113)	Per 1,000 PY	Declined high LDL-C > 160 to near 100 (*n* = 3,878)	Per 1,000 PY	*P*-value
															
	*n* (%)		*n* (%)		*n* (%)		*n* (%)		*n* (%)		*n* (%)		*n* (%)		
**Any CVD**	12,039 (8.78)	15.67	1,892 (8.96)	18.44	4,666 (8.65)	16.18	3,578 (8.51)	14.30	1,181 (8.43)	13.41	224 (10.60)	16.96	498 (12.84)	19.19	<0.0001
MI	2,590 (1.89)	3.37	337 (1.60)	3.28	963 (1.79)	3.34	789 (1.88)	3.15	290 (2.07)	3.29	57 (2.70)	4.31	154 (3.97)	5.93	<0.0001
Stroke	10,136 (7.39)	13.17	1,624 (7.69)	15.80	3,971 (7.36)	13.75	3,007 (7.15)	12.00	975 (6.96)	11.06	182 (8.61)	13.76	377 (9.72)	14.50	<0.0001
UA	835 (0.61)	1.09	124 (0.59)	1.21	308 (0.57)	1.07	236 (0.56)	0.94	75 (0.54)	0.85	32 (1.51)	2.42	60 (1.55)	2.31	<0.0001

CVD, cardiovascular disease; MI, myocardial infarction; PY, person-years; UA, unstable angina; P-value, the Chi-square test for the CVD event rate of trajectory groups.

**FIGURE 2:**
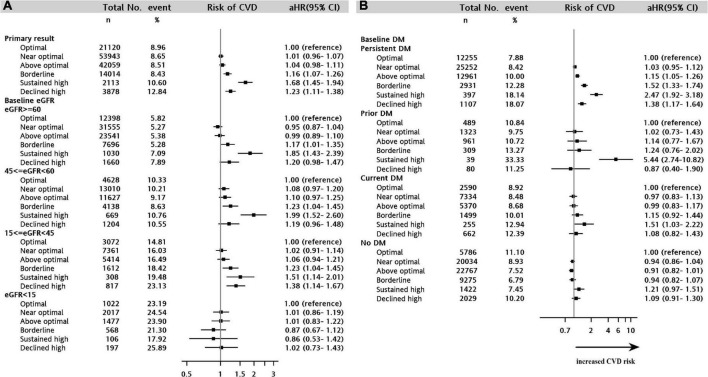
Forest plot showing the adjusted hazard ratio for cardiovascular disease among the LDL-C trajectory classes in the stratified analyses. **(A)** CKD cohort stratified by estimated glomerular filtration rate strata. **(B)** CKD cohort stratified by diabetes scenario. CKD, chronic kidney disease.

The risk of the composite CVD endpoint was higher in the borderline (aHR = 1.16, 95% CI = 1.07–1.26), sustained high (aHR = 1.68, 95% CI = 1.45–1.94), and declined high (aHR = 1.23, 95% CI = 1.11–1.38) LDL-C trajectory groups than in the optimal LDL-C trajectory group (sustained at <100 mg/dL over time) (Figure 2A and Supplementary Table 4). Overall, the association of the LDL-C trajectory with the risk of a CV event was stronger for MI than for stroke and unstable angina. Patients in the sustained high LDL-C trajectory group had higher risks of MI (aHR = 2.07, 95% CI = 1.53–2.79), unstable angina (aHR = 2.76, 95% CI = 1.8–4.25), and stroke (aHR = 1.62, 95% CI = 1.38–1.91) than those in the optimal LDL-C trajectory group (Supplementary Table 4). For the composite event of CVD, similar results were obtained from sensitivity analyses using weighted approaches in Cox model analyses with the same multiple covariates adjustment (Supplementary Table 5). These results demonstrated the robustness of observational associations between 5-year LDL-C trajectory and CVD outcomes.

### Stratified analyses

We examined whether the baseline eGFR value influenced the effect of the LDL-C trajectory on the composite CV outcome. Figure 2A shows that the aHR for the composite CV outcome decreased slightly with declining kidney function (eGFR ≥60, 45 ≤ eGFR <60, 15 ≤ eGFR <45, eGFR <15) but that the decrease was not linear. In the stratified analyses of baseline eGFR, the less optimal LDL-C trajectories (borderline, sustained high, and declined high) were consistently associated with an increased risk of CVD across the baseline eGFR strata, except when the baseline eGFR was <15 mL/min/1.73 m^2^ (all *p* > 0.05) (Figure 2A).

Differences in longitudinal changes in LDL-C were assessed in three distinct diabetes scenarios: prior (only occurring in the baseline period), current (only occurring during the follow-up period), and persistent (occurring in both the baseline and follow-up periods). A sustained high LDL-C trajectory was significantly associated with the highest increased risk of CVD in the strata of persistent diabetes (aHR = 2.47, 95% CI = 1.92–3.18), prior diabetes (aHR = 5.44, 95% CI = 2.74–10.82) and current diabetes (aHR = 1.51, 95% CI = 1.03–2.22) in Figure 2B. The less optimal LDL-C trajectories (above optimal, borderline, sustained high, and declined high) were consistently associated with an increased risk of CVD in patients with persistent diabetes (aHRs = 1.15–2.47, all *p* < 0.05). In contrast, in patients with CKD without diabetes, there was no association between the LDL-C trajectory and CVD outcome.

## Discussion

In this large CKD cohort, we categorized six distinct LDL-C trajectory patterns. We found that patients with lowered- and heightened- LDL-C trajectories throughout the 5 year follow-up were at increased risk of incident CVD. The non-linear association between LDL-C trajectories and CVD was supported when using optimal LDL-C trajectory group (near 100 mg/dL) as the reference in the Cox proportional hazard model after multivariate adjustments. Compared with the optimal group, an LDL-C level >140 mg/dL (borderline, sustained high, or declined high) accounted for an excess risk of atherosclerotic CVD of 16–68%. There was a marked difference in the effect of the LDL-C trajectory on CV outcomes according to diabetes status. Patients in the group with CKD and persistent diabetes who had a LDL-C near 120 mg/dL over time (the above optimal group) had a 15% higher risk of CVD, and this risk was greater in the borderline, sustained, and declined high trajectory groups (by 52–147%). Importantly, in the group with a high LDL-C trajectory (sustained high and declined high), a large decline in mean LDL-C attenuated the CV risk. This finding suggests that control of LDL-C reduces the risk of the composite CVD outcome in high-risk patients with CKD. Our study findings also underscore the profound effect of kidney function on the relationship between dynamic changes in LDL-C and development of atherosclerotic CVD.

Few studies have evaluated the longitudinal trend in LDL-C levels in the population with CKD. The LDL-C concentration at baseline in the present CKD cohort was lower than that in the Framingham Study cohort. It is possible that patients with CKD have multiple comorbidities and more strictly controlled lipid levels. Approximately 45.9% of participants in the Framingham Study had an LDL-C level controlled at <120 mg/dL over a 35-year observation period (14). In our CKD cohort, 84.1% of patients had an LDL-C level <120 mg/dL over time, which is similar to another Taiwanese CKD cohort in which 67.7% of patients had an LDL-C level <100 mg/dL (24). One explanation for this finding is that of 117,122 patients in our study with an optimal and near/above optimal LDL-C trajectory (baseline LDL near 120 mg/dL), the lipid-lowering treatment rate at baseline (22%) was higher than that in patients in the borderline and sustained/declined high LDL-C trajectory groups (14%). Another possible explanation is that LDL-C levels in patients with advanced CKD or end-stage kidney disease are more variable than in those with an eGFR >60 mL/min/1.73 m^2^ and in the general population (3). The characteristic lipid patterns in patients with an eGFR <45 mL/min/1.73 m^2^ are hypertriglyceridemia, low HDL-C, and high total cholesterol (25–27). The results of present study complement those of randomized clinical trials by providing longitudinal LDL-C data, which is more relevant when assessing the clinical continuum of CVD. For example, SHARP (the Study of Heart and Renal Protection) is the only clinical trial that has included a follow-up duration of more than 1 year (median 4.9 years) when evaluating the effect of lipid-lowering therapy for primary prevention of atherosclerotic CVD in patients with CKD (eGFR <60 mL/min/1.73 m^2^). SHARP showed a positive association between a high LDL-C concentration and major atherosclerotic CV events (6). The patterns of change in the LDL-C level over time identified in the present study can assist in monitoring practices for patients with CKD at higher risk of developing CVD. In view of the current absence of consensus regarding a specific LDL-C target level for lipid management in these patients, our findings provide a better understanding of when and in whom a sustained borderline or high LDL-C trajectory would be more useful than a single measurement during a short period of follow-up.

Although CKD is associated with an increased risk of CVD, the optimal LDL-C level for prevention of CVD in the CKD population remains undetermined. It has been suggested that a different CV pathology emerges in patients with an eGFR <30 mL/min/1.73 m^2^, with vascular dysfunction (stiffness and calcification), structural heart disease, and sympathetic overactivity contributing to a risk of heart failure and cardiac arrhythmia (28). Moreover, patients with end-stage kidney disease may require dialysis, which is associated with a significantly higher risk of both atherosclerotic and non-atherosclerotic CV events (1).

In line with the diverse spectrum of CKD, we found similar LDL-C trajectory patterns and their association with the risk of atherosclerotic CVD across the different baseline eGFR strata in patients with eGFR >15 mL/min/1.73 m^2^, with a slightly attenuated association in the stratum with 15 ≤ eGFR <45 mL/min/1.73 m^2^. These results are consistent with those of a study by the Alberta Kidney Disease Network that evaluated the association between baseline LDL-C category and risk of MI. In that study, the aHR for MI was weaker in patients with an LDL-C ≥4.9 mmol/L who had a lower eGFR (15–59.9 mL/min/1.73 m^2^) than in those with an eGFR of 60–89.9 mL/min/1.73 m^2^ and those with an eGFR ≥90 mL/min/1.73 m^2^ (aHR = 2.06 vs. 2.30 and 3.01, respectively) (29). The secondary analysis of SHARP showed that the association of LDL-C level with atherosclerotic vascular events was slightly weaker in patients with an eGFR <30 mL/min/1.73 m^2^ than in those with a higher eGFR (aHR = 1.13 vs. 1.23) (30). Because the sample size was small in the strata of eGFR <15 mL/min/1.73 m^2^, the association between LDL-C trajectory and CVD risk needs to be examined in further research.

Some traditional risk factors for CVD, such as diabetes, dyslipidemia, and hypertension, are common in patients with CKD (31), and CVD is the most common complication in patients with diabetes (32). The Framingham Heart Study showed that diabetes increased the risk of CVD by 2–3-fold (33). A previous observational study also indicated a positive linear association between the LDL-C level and the risk of major adverse CV events in patients with CKD and diabetes (13). There was considerable variation in the LDL-C trajectory between patients with and without baseline diabetes (Figures 1B,C), whereas the intercept was lower in patients with baseline diabetes, which might be related to baseline pharmacotherapy for lipid control. In addition, we found that diabetes enhanced the association between a high LDL-C trajectory and the risk of CVD (Figure 2B). These study findings suggested that patients with CKD and diabetes should be offered at least annual monitoring of LDL-C levels to avoid underdiagnosed dyslipidemia or undertreated with lipid-lowering therapy. Clinicians should consider a stricter LDL-C control strategy, such as near 100–120 mg/dL over time, which may be beneficial in high risk of patients with CKD and diabetes.

This study has several strengths, in particular its large-scale nature and use of valuable clinical data on longitudinal changes in LDL-C in patients with a long follow-up duration. To the best of our knowledge, this is the first study to investigate the heterogeneity of changes in LDL-C in the CKD population over a long period of time according to diabetes status. Furthermore, we used a latent class growth mixed model to identify variations in different LDL-C categories over time in a general CKD cohort, taking into account all levels of kidney disease.

There were also some limitations to this study. First, the LDL-C trajectory model was fitted using the LDL-C value for 5 years of follow-up rather than all available measurements. Changes in LDL-C within a short period of time (e.g., within 6 months) or after 5 years of follow-up could have been missed and introduced some degree of bias. Further investigation is needed to confirm the association between LDL-C trajectory and CVD outcomes. Second, CKD-specific risk factors, such as malnutrition, inflammation, oxidative stress, and uremic toxins, which contribute to CV events and mortality (34), were not measured. Moreover, obesity, smoking, lifestyle factors, diet, and family history of diseases were not included. Future studies should focus on the above-mentioned risk factors in patients with CKD to minimize potential confounders. Third, although this study was performed using CGRD, which is the largest multi-institutional electronic health record dataset in Taiwan, the generalizability of our findings might still be limited. Ethnic differences and genetic variations are associated with the distribution of lipoproteins and the level of CV risk (35). Our study population might be representative of Taiwanese and even Asian populations, but caution is needed when generalizing our data to other ethnic groups.

In summary, this study found that certain longitudinal changes in LDL-C were associated with an increased risk of composite CV events in patients with any stage of CKD except in those with an eGFR <15 mL/min/1.73 m^2^. This finding suggests that there is no difference in the risk of atherosclerotic CVD between an LDL-C level near 120 mg/dL and an LDL-C <100 mg/dL over time in patients with CKD and an eGFR ≥15 mL/min/1.73 m^2^. Diabetes increases the strength of the association between the LDL-C level and risk of CVD, and improvements in LDL-C over time (about 100 mg/dL) may be beneficial for CKD patients with diabetes. Future research is warranted to investigate the optimal target LDL-C levels which may help to identify patients who require intensive individualized lipid management.

## Data availability statement

The original contributions presented in this study are included in the article/Supplementary material, further inquiries can be directed to the corresponding author/s.

## Author contributions

S-WW and C-NH: conceptualization and wrote manuscript—original draft preparation. S-WW and H-CK: formal analysis and methodology. S-WW: funding acquisition. All authors: write manuscript—review and editing, investigation, validation, and visualization.
