# Supplementary material for: Trajectory of low-density lipoprotein cholesterol in patients with chronic kidney disease and its association with cardiovascular disease
Source: Front Cardiovasc Med. 2022 Jul 26;9:887915. doi: 10.3389/fcvm.2022.887915 (PMC9360605; doi:10.3389/fcvm.2022.887915)

**Trajectory of low-density lipoprotein cholesterol in patients with chronic kidney disease and its association with cardiovascular disease**

**SUPPLEMENTARY MATERIAL**

Supplementary Table 1. Results of the fitting process using latent class growth mixed models (LCGMM)

Supplementary Table 2. Trajectory model fit diagnostics

Supplementary Table 3. Linear mixed model for changes in LDL-C during follow-up

Supplementary Table 4. Crude and adjusted hazard ratios for composite CVD, MI, stroke, and unstable angina in each trajectory class

Supplementary Table 5. Association between the risk of composite CVD and LDL-C trajectory using weighted approaches

Supplementary Figure 1. Flow chart showing the patient selection process.

Supplementary Figure 2. Predicted annual changes in LDL-C levels in patients with CKD according to diabetes status.

**Supplementary Table 1.** Results of the fitting process using latent class growth mixed models (LCGMM)

| No. of latent classes | Polynomial degree | Log-Lik | BIC |  | % Participants per class | Mean posterior probabilities |
| --- | --- | --- | --- | --- | --- | --- |
| 3 | Linear | -2474198.539 | -2474257.914 |  | 39.22/48.67/12.11 | 0.8887/0.8643/0.8779 |
|  | Quadratic | -2473162.622 | -2473241.788 |  | 39.35/48.58/12.07 | 0.8894/0.8640/0.8772 |
|  | Cubic | -2472978.725 | -2473077.683 |  | 39.31/48.62/12.08 | 0.8894/0.8641/0.8776 |
| 4 | Linear | -2465479.299 | -2465558.466 |  | 25.69/44.94/25.30/ 4.07 | 0.8521/0.8075/0.8302/0.8766 |
|  | Quadratic | -2464386.484 | -2464492.040 |  | 25.74/44.94/25.24/ 4.08 | 0.8518/0.8077/0.8303/0.8766 |
|  | Cubic | -2464200.242 | -2464332.186 |  | 25.73/44.97/25.23/ 4.07 | 0.8520/0.8078/0.8305/0.8772 |
| 5 | Linear | -2462228.005 | -2462326.963 |  | 17.14/38.54/31.82/10.92/ 1.57 | 0.8155/0.7658/0.7730/0.8056/0.8742 |
|  | Quadratic | -2461096.145 | -2461228.089 |  | 17.24/38.69/31.71/10.82/ 1.56 | 0.8162/0.7670/0.7741/0.8058/0.8770 |
|  | Cubic | -2460906.687 | -2461071.617 |  | 17.12/38.62/31.79/10.90/ 1.57 | 0.8155/0.7668/0.7738/0.8060/0.8762 |
| **6** | Linear | -2458711.252 | -2458830.002 |  | 16.97/37.57/29.59/10.22/ 4.04/ 1.61 | 0.8211/0.7648/0.7445/0.7543/0.7256/0.8693 |
|  | Quadratic | -2456772.885 | -2456931.218 |  | 16.79/37.56/29.94/10.69/ 3.40/ 1.61 | 0.8219/0.7686/0.7576/0.7866/0.7491/0.8751 |
|  | **Cubic** | **-2456526.385** | **-2456724.301** |  | **16.67/37.45/29.98/10.75/ 1.64/ 3.52** | **0.8212/0.7681/0.7566/0.7858/0.8783/0.7493** |
| 7 | Linear | -2456533.888 | -2456672.429 |  | 15.18/ 1.89/35.62/29.25/ 5.16/12.03/ 0.87 | 0.8159/0.7924/0.7561/0.7210/0.7088/0.7378/0.8448 |
|  | Quadratic | -2454414.962 | -2454599.684 |  | 16.34/ 1.55/36.11/29.88/10.54/ 4.05/ 1.52 | 0.8220/0.8438/0.7638/0.7554/0.7703/0.7171/0.7833 |
|  | Cubic | -2454857.569 | -2455088.471 |  | 13.17/33.19/30.73/15.13/ 3.65/ 3.68/ 0.45 | 0.8045/0.7435/0.7194/0.7394/0.8155/0.7388/0.8918 |

Six latent classes with cubic terms were chosen and defined as optimal LDL-C (<100 mg/dL; 16.67%), near optimal LDL-C (near 100 mg/dL; 37.45%), above optimal LDL-C (near 120 mg/dL; 29.98%), borderline LDL-C (near 140 mg/dL; 10.75%), sustained high LDL-C (>160 mg/dL; 1.64%), and declined high LDL-C (drop from >160 to <100 mg/dL; 3.52%) based on the National Cholesterol Education Program Adult Treatment Panel III classification criteria for LDL-C level. BIC, Bayesian information criterion; Log-Lik, Log-likelihood

SAS syntax of latent class growth mixture model (LCGMM)

**PROC** **TRAJ** DATA=ANAL OUTPLOT=OP OUTSTAT=OS OUT=OF OUTEST=OE ITDETAIL altstart;

ID IDCODE; VAR LDL0-LDL5; INDEP TIME0-TIME5;

MODEL CNORM; MAX **1000**; NGROUPS **6**; ORDER **3** **3** **3** **3** **3** **3**;

**RUN**;

Reference: JONES BL, NAGIN DS, ROEDER K. A SAS Procedure Based on Mixture Models for Estimating Developmental Trajectories. (2001) 29(3):374-93.

**Supplementary Table 2.** Trajectory model fit diagnostics

| Latent class | n | Mean posterior probabilities (mPP) | % Participants per class (π) | OCC |
| --- | --- | --- | --- | --- |
| Optimal  (LDL-C<100 mg/dL) | 21120 | 0.8212 | 0.1667 | 23 |
| Near optimal  (LDL-C near 100 mg/dL) | 53943 | 0.7681 | 0.3745 | 6 |
| Above optimal  (LDL-C near 120 mg/dL) | 42059 | 0.7566 | 0.2998 | 7 |
| Borderline  (LDL-C near 140 mg/dL) | 14041 | 0.7858 | 0.1075 | 30 |
| Sustained high  (LDL-C>160 mg/dL) | 2113 | 0.8783 | 0.0164 | 434 |
| Declined high  (LDL-C >160 to near 100 mg/dL ) | 3878 | 0.7493 | 0.0352 | 82 |

Odds of correct classification (OCC) per class is defined as$=\frac{\frac{mPP}{(1-mPP)}}{\frac{\pi}{(1-\pi)}}$;

Higher OCCs indicate a better fitting model and an OCC >5 for all groups suggests good assignment accuracy.

Reference: (24) Nagin D. Group-based modeling of development. Cambridge, MA: Harvard University Press; 2005.

**Supplementary Table 3.** Linear mixed model for changes in LDL-C during follow-up

|  | | Model 1 | | | | |  | Model 2 | | | | |
| --- | --- | --- | --- | --- | --- | --- | --- | --- | --- | --- | --- | --- |
|  |  | Adjusted β | 95% CI | | | *P* value |  | Adjusted β | 95% CI | | | *P* value |
| Intercept | | 73.56 | (73.39 | , | 73.72) | <.0001 |  | 77.33 | (77.02 | , | 77.64) | <.0001 |
| **LDL-C trajectory group** | |  |  |  |  |  |  |  |  |  |  |  |
|  | Optimal | (Reference) | | | |  |  | (Reference) | | | |  |
|  | Near optimal | 24.31 | (24.13 | , | 24.50) | <.0001 |  | 24.01 | (23.83 | , | 24.18) | <.0001 |
|  | Above optimal | 45.12 | (44.92 | , | 45.32) | <.0001 |  | 44.53 | (44.34 | , | 44.72) | <.0001 |
|  | Borderline | 69.42 | (69.15 | , | 69.68) | <.0001 |  | 68.61 | (68.36 | , | 68.86) | <.0001 |
|  | Sustained high | 105.91 | (105.32 | , | 106.51) | <.0001 |  | 104.95 | (104.40 | , | 105.51) | <.0001 |
|  | Declined high | 82.09 | (81.66 | , | 82.53) | <.0001 |  | 81.01 | (80.61 | , | 81.42) | <.0001 |
| **Follow-up time (year)** | | -1.63 | (-1.70 | , | -1.56) | <.0001 |  | -1.49 | (-1.55 | , | -1.42) | <.0001 |
| **LDL-C trajectory group*Follow-up time (year)** | | | | | | | | | | | | |
|  | Optimal*time | (Reference) | | | |  |  | (Reference) | | | |  |
|  | Near optimal*time | -0.70 | (-0.78 | , | -0.62) | <.0001 |  | -0.77 | (-0.84 | , | -0.69) | <.0001 |
|  | Above optimal*time | 0.47 | (0.39 | , | 0.55) | <.0001 |  | 0.27 | (0.19 | , | 0.35) | <.0001 |
|  | Borderline*time | 0.58 | (0.47 | , | 0.68) | <.0001 |  | 0.34 | (0.23 | , | 0.44) | <.0001 |
|  | Sustained high*time | -0.57 | (-0.81 | , | -0.34) | <.0001 |  | -0.88 | (-1.11 | , | -0.64) | <.0001 |
|  | Declined high*time | -15.28 | (-15.44 | , | -15.12) | <.0001 |  | -15.34 | (-15.50 | , | -15.18) | <.0001 |
| **Sex (Male vs. Female)** | |  |  |  |  |  |  | -0.44 | (-0.52 | , | -0.36) | <.0001 |
| **Age at index date** | |  |  |  |  |  |  | -0.03 | (-0.04 | , | -0.03) | <.0001 |
| **Baseline eGFR** | |  |  |  |  |  |  | -0.01 | (-0.01 | , | 0.00) | <.0001 |
| **Baseline DM (yes vs. no)** | |  |  |  |  |  |  | -1.57 | (-1.65 | , | -1.48) | <.0001 |
| **BIC (** Bayesian information criterion) | | 12116222 |  |  |  |  |  | 12124389 |  |  |  |  |

**Supplementary Table 4**. Crude and adjusted hazard ratios for composite CVD, MI, stroke, and unstable angina in each trajectory class

|  | **Composite CVD** | | | | | | | |  | | **MI** | | | | | | | |  | | **Stroke** | | | | | | | |  | | **Unstable angina** | | | | | | | |  |
| --- | --- | --- | --- | --- | --- | --- | --- | --- | --- | --- | --- | --- | --- | --- | --- | --- | --- | --- | --- | --- | --- | --- | --- | --- | --- | --- | --- | --- | --- | --- | --- | --- | --- | --- | --- | --- | --- | --- | --- |
| **LDL trajectory*** | | **Crude HR** | | **95% CI** | | | | *p-value* | |  | | **Crude HR** | | **95% CI** | | | | *p-value* | |  | | **Crude HR** | | **95% CI** | | | | *p-value* | |  | | **Crude  HR** | | **95% CI** | | | | *p-value* | |
| Optimal | 1.00 | | (reference) | | | |  | |  | | 1.00 | | (reference) | | | |  | |  | | 1.00 | | (reference) | | | |  | |  | | 1.00 | | (reference) | | | |  | |  |
| Near optimal | 0.83 | | (0.79 | |  | 0.88) | <.0001 | |  | | 0.96 | | (0.85 | |  | 1.09) | 0.5475 | |  | | 0.82 | | (0.77 | |  | 0.87) | <.0001 | |  | | 0.86 | | (0.69 | |  | 1.05) | 0.1406 | |  |
| Above optimal | 0.70 | | (0.66 | |  | 0.74) | <.0001 | |  | | 0.86 | | (0.76 | |  | 0.98) | 0.0215 | |  | | 0.67 | | (0.63 | |  | 0.71) | <.0001 | |  | | 0.73 | | (0.59 | |  | 0.91) | 0.0049 | |  |
| Borderline | 0.64 | | (0.59 | |  | 0.69) | <.0001 | |  | | 0.88 | | (0.75 | |  | 1.03) | 0.1016 | |  | | 0.60 | | (0.56 | |  | 0.65) | <.0001 | |  | | 0.65 | | (0.49 | |  | 0.87) | 0.0036 | |  |
| Sustained high | 0.80 | | (0.70 | |  | 0.92) | 0.0019 | |  | | 1.14 | | (0.86 | |  | 1.51) | 0.3657 | |  | | 0.74 | | (0.63 | |  | 0.86) | 0.0001 | |  | | 1.87 | | (1.27 | |  | 2.76) | 0.0016 | |  |
| Declined high | 0.89 | | (0.81 | |  | 0.99) | 0.0233 | |  | | 1.54 | | (1.27 | |  | 1.86) | <.0001 | |  | | 0.76 | | (0.68 | |  | 0.85) | <.0001 | |  | | 1.75 | | (1.29 | |  | 2.39) | 0.0004 | |  |
|  | | **aHR**** | | **95% CI** | | | | *p-value* | |  | | **aHR** | | **95% CI** | | | | *p-value* | |  | | **aHR** | | **95% CI** | | | | *p-value* | |  | | **aHR** | | **95% CI** | | | | *p-value* | |
| Optimal | 1.00 | | (reference) | | | |  | |  | | 1.00 | | (reference) | | | |  | |  | | 1.00 | | (reference) | | | |  | |  | | 1.00 | | (reference) | | | |  | |  |
| Near optimal | 1.01 | | (0.96 | |  | 1.07) | 0.7500 | |  | | **1.16** | | **(1.02** | |  | **1.32)** | **0.0237** | |  | | 1.00 | | (0.94 | |  | 1.06) | 0.9746 | |  | | 0.97 | | (0.78 | |  | 1.20) | 0.7595 | |  |
| Above optimal | 1.04 | | (0.98 | |  | 1.11) | 0.2061 | |  | | **1.28** | | **(1.11** | |  | **1.48)** | **0.0006** | |  | | 1.02 | | (0.95 | |  | 1.09) | 0.5890 | |  | | 0.96 | | (0.75 | |  | 1.22) | 0.7146 | |  |
| Borderline | **1.16** | | **(1.07** | |  | **1.26)** | **0.0006** | |  | | **1.50** | | **(1.25** | |  | **1.80)** | **<.0001** | |  | | **1.13** | | **(1.03** | |  | **1.24)** | **0.0111** | |  | | 0.94 | | (0.68 | |  | 1.30) | 0.6910 | |  |
| Sustained high | **1.68** | | **(1.45** | |  | **1.94)** | **<.0001** | |  | | **2.07** | | **(1.53** | |  | **2.79)** | **<.0001** | |  | | **1.62** | | **(1.38** | |  | **1.91)** | **<.0001** | |  | | **2.76** | | **(1.80** | |  | **4.25)** | **<.0001** | |  |
| Declined high | **1.23** | | **(1.11** | |  | **1.38)** | **0.0002** | |  | | **1.66** | | **(1.34** | |  | **2.05)** | **<.0001** | |  | | 1.12 | | (0.99 | |  | 1.27) | 0.0640 | |  | | **1.61** | | **(1.13** | |  | **2.29)** | **0.0080** | |  |

*Optimal LDL-C (<100 mg/dL; 16.67%), near optimal LDL-C (near 100 mg/dL; 37.45%), above optimal LDL-C (near 120 mg/dL; 29.98%), borderline LDL-C (near 140 mg/dL; 10.75%), sustained high LDL-C (>160 mg/dL; 1.64%), and declined high LDL-C (drop from >160 to <100 mg/dL; 3.52%).

**The adjusted hazard ratio (aHR) was estimated in a Cox proportional hazards model with adjustment for age, sex, baseline LDL-C, baseline CKD stage, comorbidities, prior medication, and concomitant medication (lipid-lowering agent, antidiabetic agent, antihypertensive agent, antiplatelet, anticoagulant).

CVD, cardiovascular disease; MI, myocardial infarction; UA, unstable angina

**Supplementary Table 5.** Association between the risk of composite CVD and LDL-C trajectory using weighted approaches

|  | | **Primary Cox model** | | | | |  | | | | | **Weighted Cox model^#^** | | | | | |
| --- | --- | --- | --- | --- | --- | --- | --- | --- | --- | --- | --- | --- | --- | --- | --- | --- | --- |
| LDL-C trajectory* | aHR** | | 95% CI | | | *p-value* | | |  | | aHR** | | | 95% CI | | | *p-value* |
| Optimal | | 1.00 | (reference) | | |  | |  | | 1.00 | | | (reference) | | | |  |
| Near optimal | | 1.01 | (0.96 |  | 1.07) | 0.7500 | |  | | 1.00 | | | (0.94 | |  | 1.05) | 0.8604 |
| Above optimal | | 1.04 | (0.98 |  | 1.11) | 0.2061 | |  | | 1.03 | | | (0.96 | |  | 1.10) | 0.4419 |
| Borderline | | **1.16** | **(1.07** |  | **1.26)** | **0.0006** | |  | | 1.17 | | | (1.07 | |  | 1.28) | 0.0004 |
| Sustained high | | **1.68** | **(1.45** |  | **1.94)** | **<.0001** | |  | | 1.72 | | | (1.47 | |  | 2.01) | <.0001 |
| Declined high | | **1.23** | **(1.11** |  | **1.38)** | **0.0002** | |  | | 1.25 | | | (1.12 | |  | 1.40) | 0.0001 |

*Optimal LDL-C (<100 mg/dL; 16.67%), near optimal LDL-C (near 100 mg/dL; 37.45%), above optimal LDL-C (near 120 mg/dL; 29.98%), borderline LDL-C (near 140 mg/dL; 10.75%), sustained high LDL-C (>160 mg/dL; 1.64%), and declined high LDL-C (drop from >160 to <100 mg/dL; 3.52%).

**The adjusted hazard ratio (aHR) was estimated in all Cox proportional hazards model with adjustment for age, sex, baseline LDL-C, baseline CKD stage, comorbidities, prior medication, and concomitant medication (lipid-lowering agent, antidiabetic agent, antihypertensive agent, antiplatelet, anticoagulant).

^#^ Weighted Cox model: weight was based on the each patient’s posterior probability in the specific LDL-C trajectory group.

Composite CVD (cardiovascular disease) includes myocardial infarction, stroke, and unstable angina.

**Supplementary Figure 1.** Flowchart of patient selection process

Newly-diagnosed chronic kidney disease patients with ≥3 LDL-C measures, January 1, 2004 and December 31, 2018

(N=177,828)

LDL-C trajectory cohort

(N=137,127)

**Exclusions (N=40,701)**

1. No SCr value before index date <1 year (n=7,422)

2. With past history as following:

- Myocardial infarction (n=5,679)
- Unstable angina (n=3,424)
- Ischemic stroke (n=26,594)
- Coronary artery bypass graft (CABG) (n=925)
- Percutaneous coronary intervention (PCI) (n=6,593)

**Near optimal**

(LDL-C near 100)

**Above optimal**

(LDL-C near 120)

**Borderline**

(LDL-C near 140)

**Sustained high**

(LDL-C >160)

**Declined high**

(LDL-C >160 to near 100)

**Optimal**

(LDL-C<100)

**Supplementary Figure 2. Predicted annual changes in LDL-C levels in patients with CKD according to diabetes status.**


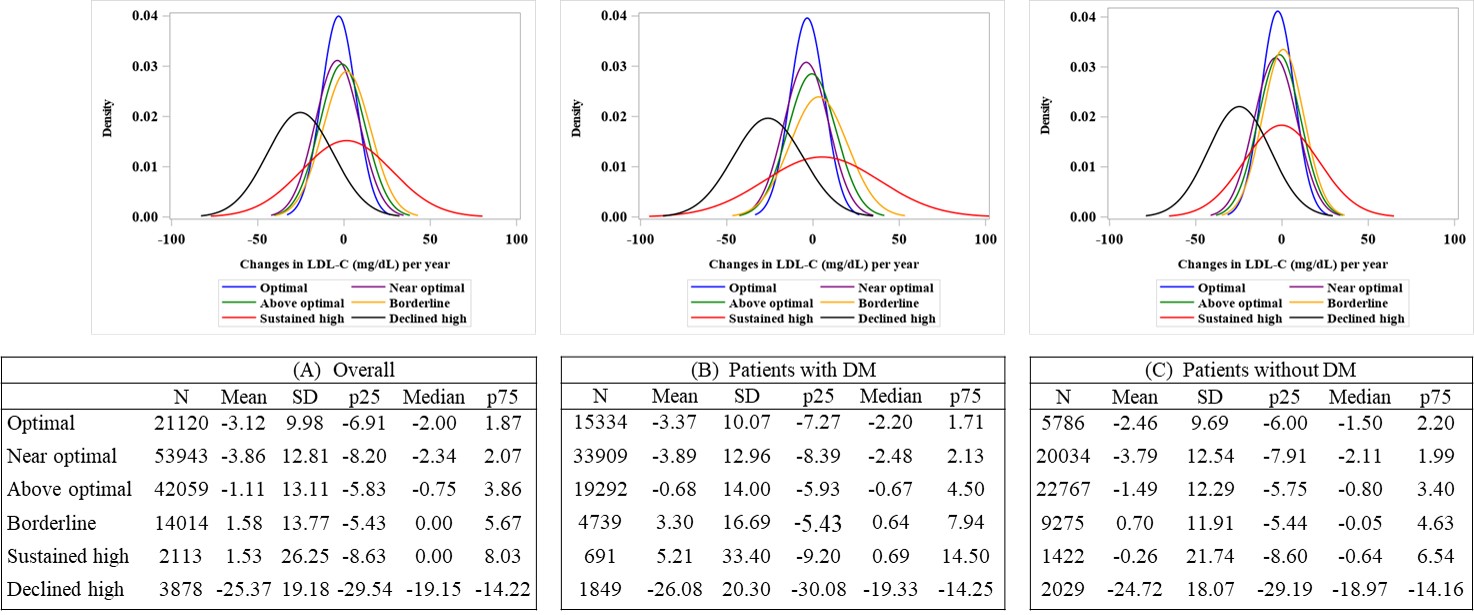

Supplement: Supplementary file 1 [file Data_Sheet_1.docx]
